# Circ_ 0115744 acts as miR-144 sponge to promote and predict the metastasis of colorectal cancer

**DOI:** 10.18632/aging.202513

**Published:** 2021-02-11

**Authors:** Xiaoming Ma, Long Lv, Chungen Xing

**Affiliations:** 1 Department of General Surgery, The Second Affiliated Hospital of Soochow University, Suzhou 215004, China; 2 Department of General Surgery, The Affiliated Suqian Hospital of Xuzhou Medical University, Suqian 223800, China; 3 Department of General Surgery, Gaochun People Hospital, Gaochun 210000, China

**Keywords:** circRNA, colorectal cancer, liver metastasis, miRNA, ceRNA

## Abstract

Colorectal cancer (CRC) is one of the most common human malignant tumors in recent years. Although multiple approaches have been developed for the diagnosis and therapy of CRC, the overall survival rates of patients with metastatic and recurrent CRC remain poor. In the present study, we used the high-throughput microarray technology to screen circular RNAs (circRNAs) as a potential fingerprint for CRC. We mainly aimed to screen potential biomarkers for liver metastasis by performing risk score analysis. We detected the upregulated expression of circ_0115744 in patients with CRC with liver metastasis (CRLM). Further investigation using a validation set indicated that circ_0115744 might be considered as a fingerprint for CRLM. Functionally, the overexpression of circ_0115744 significantly promoted the invasion of CRC cell lines, while decreased expression of circ_0115744 suppressed cell invasion *in vitro*. Mechanistic analysis showed that circ_0115744 acted as a competing endogenous RNA of miR-144 to diminish the repressive effect of miR-144 on its target EZH2. In conclusion, we found that increased expression of circ_0115744 could differentiate CRLM from CRC and that the newly identified circ_0115744/miR-144/EZH2 axis was involved in the progression of CRC, which might be used as a potential diagnostic and therapeutic target for patients with CRC.

## INTRODUCTION

According to the latest cancer statistics report in 2020, colorectal cancer (CRC) is one of the most common malignancies of the gastrointestinal tract in humans, with the third incidence rate in males and the second one in females [1]. In China, with the improvement in people’s living standards and diet structure, the incidence and mortality of CRC are increasing rapidly [2]. In the past decades, surgery, chemotherapy, radiotherapy, and immunotherapy have been developed, which highly increased the survival of patients with CRC [3]. However, the overall survival of patients with CRC with liver metastasis (CRLM) remains poor [4, 5]. Previous studies have shown that approximately 15%–25% of patients with CRC have synchronous liver metastasis [6]. Moreover, even though neoadjuvant therapy has gained widespread popularity, the prognosis of these patients remains a challenge. For CRC with liver metastasis, traditional biomarkers such as CEA and CA19-9 were shown to have low sensitivity and specificity in clinical studies [7]. Thus, the early diagnosis and therapy of CRC have become an urgent and crucial topic of research.

Circular RNAs (circRNAs) are a new class of noncoding RNAs that form covalently closed continuous loops without terminal 5ʹ-caps and 3ʹ-polyadenylated tails [8]. They are generated by back-splicing of pre-mRNA transcripts, in which an upstream splice acceptor is linked to a downstream splice donor [9]. Because of the closed loop structure, circRNAs are not easily degraded by the exonuclease RNase R and show greater stability than linear RNAs [10]. On the basis of these features, circRNAs are defined as abundant, stable, and conserved molecules and usually exhibit tissue or developmental stage-specific expression [11]. CircRNA was found to participate in multiple human diseases through different approaches. It was observed that circRNAs possess protein translation ability and could also be regarded as efficient miRNA sponges, as they contain conserved miRNA target sites [12]. Because of the stable expression and tissue-specific characteristics of circRNAs, they are found to be ideal biomarkers for the diagnosis and prognosis of cancers. For example, tissue circRNA0003906 and hsa_circ_0004585 were reported as biomarkers for CRC [13, 14].

In the present study, we aimed to screen potential circRNAs as biomarkers for predicting liver metastasis in patients with CRC. We first evaluated the expression profiles of circRNAs in plasma samples of 10 patients with CRC with liver metastasis (CRLM), 10 patients with CRC without liver metastasis (CRC), and healthy controls by microarrays, and identified that circ_0115744 was significantly upregulated in the CRLM group compared to that in the CRC and control groups. The risk score analysis revealed that circ_0115744 could predict CRLM from the CRC and control groups. The results of bioinformatics prediction analysis implied that circ_0115744 acts as the sponge of miR-144 in CRC cells and that it might promote metastasis through an miR-144-depedent mechanism. The subsequent experiments also suggested that EZH2 is the target of miR-144. Our findings indicated that circRNAs might exert regulatory functions in CRC and that circ_0115744 might serve as a biomarker for predicting CRLM.

## RESULTS

### Identification of circRNA landscape in CRC with liver metastasis

**Figure 1:**
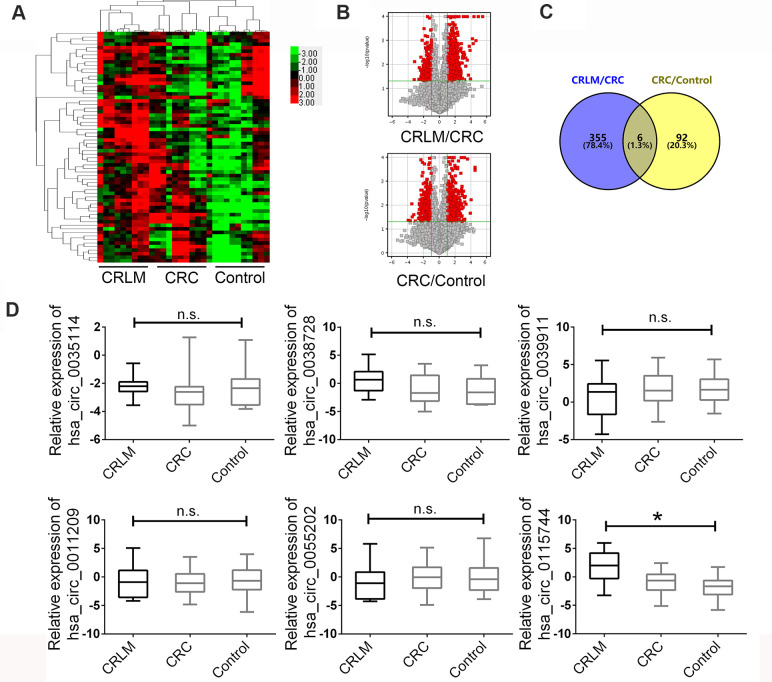
**The expression landscape of circulating circRNAs in HCC patients and matched control groups.** (**A**) Cluster analysis of the different expression of the circRNAs extracted from plasma in different groups. (**B**) Volcano plot shows the up-regulated and down-regulated circRNAs in different groups. Higher expression levels are indicated by “red” and no significant difference is indicated by “black”. (**C**) Venny map for upregulated circRNAs in CRLM group comparing with CRC and CRC group comparing with healthy control group. (**D**) The expression of circRNAs were confirmed by RT-PCR in groups. Data were presented as plot of the mean with SEM with log-transformed. CRLM: colorectal cancer with liver metastasis; CRC: colorectal cancer without liver metastasis. *Indicated P < 0.05, n.s. indicated no significant.

The circRNA microarray was used to detect the circRNA expression landscape in plasma samples of 10 newly diagnosed patients with CRC with liver metastasis (CRLM group), matched plasma samples of 10 patients with CRC without liver metastasis (CRC group), and plasma samples of 10 normal individuals as the control group. Clustering analysis with a heat map showed different expression profiles of these three groups (Figure 1A). A paired comparison was used to analyze statistically significant circRNAs in the CRLM group. Patients with CRC were compared with healthy controls by using volcano plots with the following parameters: fold changes ≥2.0 and p-value ≤ 0.05. As shown in Figure 1B, the results indicated a remarkably different expression landscape of circRNAs in the three groups. Next, we analyzed the upregulated circRNAs in the CRLM group compared to that in the CRC group and the increased circRNAs in CRC group comparing with healthy controls. The Venny map indicated that among the dysregulated circRNAs, six circRNAs showed a rapid increase in their expression level in plasma samples of the control, CRC, and CRLM groups (Figure 1C).

Next, a large-scale sample size was used to measure the different expression levels of the six candidate circRNAs in plasma samples as the validation set. A total of 190 patients diagnosed with CRLM, 190 patients diagnosed with CRC without liver metastasis, and 190 healthy controls were enrolled. The clinicopathological features of the enrolled subjects are presented in Table 1, and the age and gender were matched. After the detection of the six circRNAs in the plasma samples mentioned above, we found that circ_0115744 showed consistent CHIP expression in all three groups, hsa_circ_0035114 and hsa_circ_0038728 showed no significant expression in the control group, hsa_circ_0039911 and hsa_circ_0055202 were contrary to the chip expression in the CRLM and CRC groups, and hsa_circ_0011209 showed no significant expression in all three groups (Figure 1D).

**Table 1:** Clinicopathological features of colorectal cancer (CRC) and cancer-free control samples.

**N**	**CRLM**	**CRC**	**Control**	***P* valve**
Age Mean (SE) year	55.33(0.11)	54.28(0.41)	56.19(0.43)	0.45^a^
Sex (male/female)	135/65	122/78	134/66	0.32^b^
**Differentiation grade**				
Well	0	0		0.19^b^
Moderate	144	132		
Poorly	56	68		
**Tumor Size(cm)**				0.61^b^
≤5 cm	127	122		
>5 cm	73	78		
**cT staging system**				0.16^b^
T1+T2	89	75		
T3+T4	111	125		

^a^Student t-test.

^b^Chi-square test.

### Diagnostic potency prediction by risk score analysis

**Figure 2:**
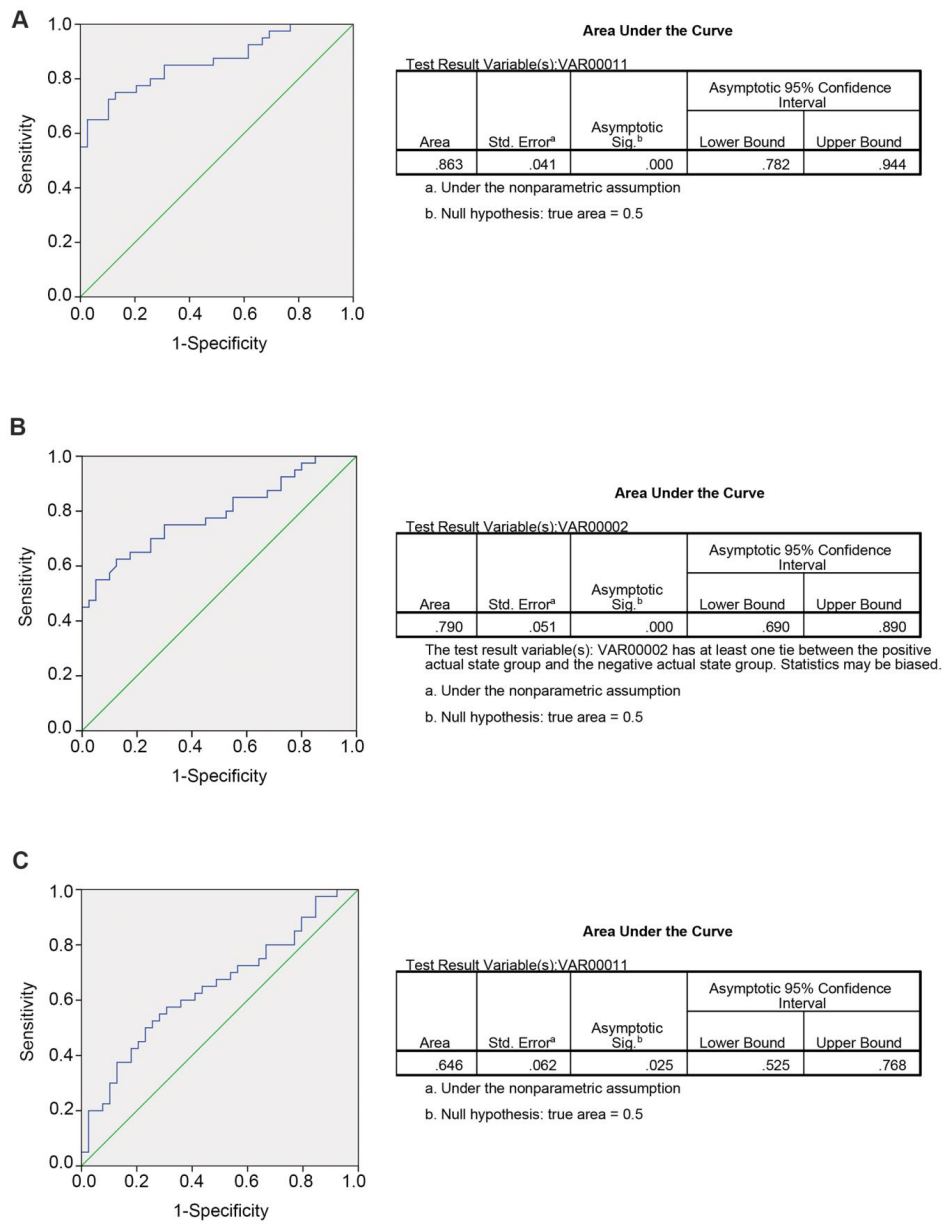
**Predicting ability for candidate circRNA in validation set.** The ROC curve for circRNA fingerprint in validation set. (**A**) CRLM comparing with CRC; (**B**) CRC comparing with control group; (**C**) CRLM comparing with control group.

Researchers have identified that circRNAs might serve as potential biomarkers for human malignant tumors; however, they focused only on the predictive value of circRNAs in tissues samples, and little evidence was provided for these circRNAs to predict CRLM from patients with CRC or healthy controls in plasma samples. Next, to further examine the detailed accuracy and specificity of circ_0115744 as a CRLM potential signature, the risk score formula was used. First, circ_0115744 was analyzed to detect whether it could predict CRLM from CRC. In the risk score analysis, the training set was divided into a higher risk group and a lower risk group, which indicating the CRLM group and CRC group. Next, the cutoff value defined as the maximal value for sensitivity + specificity was used. We applied the cutoff value of 7.911, and the positive predictive value (PPV) and negative predictive value (NPV) obtained in the training set were 80% and 80%, respectively. The same method was used in the validation set, and the PPV and NPV were 94% and 94%, respectively (Table 2). The areas under the ROC curves (AUC) of circ_0115744 was 0.863 (Figure 2A). We next measured the ability for circ_0115744 to predicting CRC from the control group. The PPV and NPV were 80% and 80% in the training set, and 94% and 94% in the validation set, respectively (Table 2), and the AUC was 0.790 (Figure 2B). The predictive ability for CRLM from the control group was also detected. The PPV and NPV were 90% and 90% in the training set and 78% and 73% in the validation set, respectively (Table 2), and the AUC was 0.646 (Figure 2C).

**Table 2:** Risk score analysis of CRLM and CRC control plasma samples.

**Score**	0-7.911	7.91-14.43	PPV	NPV
**Training set**			0.80	0.80
CRLM	2	8		
CRC	8	2		
**Validation set**			0.94	0.94
CRLM	12	178		
CRC	178	12		
**Score**	0–5.362	5.362–9.112	PPV	NPV
**Training set**			0.80	0.80
CRC	8	2		
Control	2	8		
**Validation set**			0.91	0.91
CRC	18	172		
Control	172	18		
**Score**	0–4.731	4.731-6.889	PPV	NPV
**Training set**			0.90	0.90
CRLM	1	9		
Control	9	1		
**Validation set**			0.78	0.73
CRLM	37	153		
Control	134	56		

PPV, positive predictive value.

NPV, negative predictive value.

### Increased circ_0115744 expression promoted invasion of CRC

**Figure 3:**
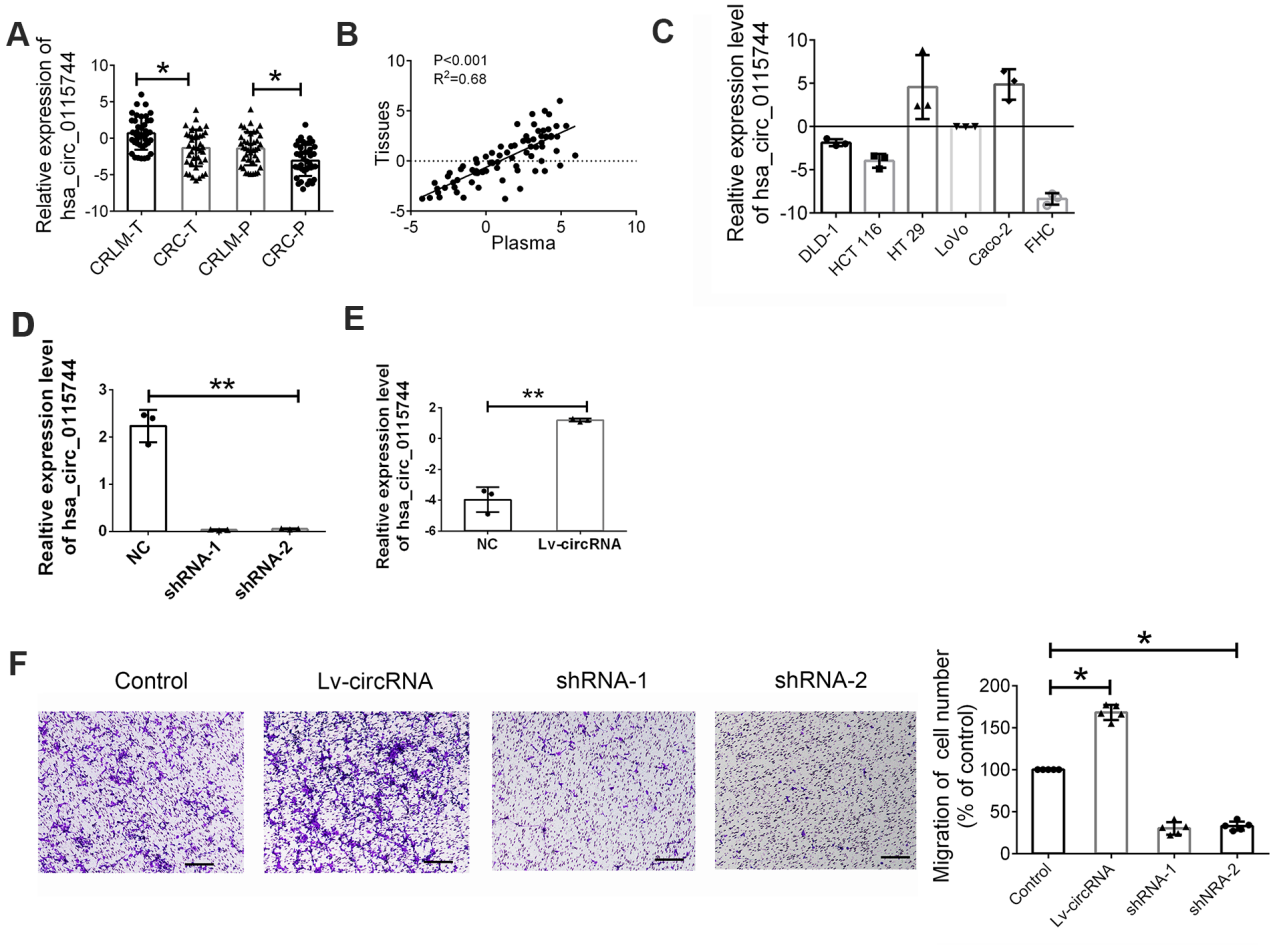
**Increased circ_0115744 promoted cell invasion of CRC.** (**A**) Relative expression of circ_0115744 in tissues samples of CRLM and CRC. Data were presented as plot of the mean with SD with log-transformed. (**B**) Pearson correlation analysis for circ_0115744 in tissues and plasma. (**C**) Relative expression of circ_0115744 in CRC cell lines. (**D**) Relative expression of circ_0115744 in cells treated with shRNA lentivirus. (**E**) Relative expression of circ_0115744 in cells treated with overexpression lentivirus. (**F**) Cell invasion in DLD-1 cells. *Indicated P < 0.05, ** indicated P < 0.01.

As the function of circ_0115744 is unclear in human cancers, we next detected whether circ_0115744 was abnormally expressed in the tissues samples of patients. with CRLM or corrected with circulating expression Among the 200 patients with CRLM and CRC, 40 patients received resection for CRC and synchronous liver metastasis node. Furthermore, randomly selected 40 CRC patients who also received resection for CRC but were without liver metastasis were also enrolled. First, we analyzed the expression of circ_0115744 in tumor tissues and the corresponding adjacent tissues. As shown in Figure 3A, we found that circ_0115744 expression was increased in tumor tissues of both the CRLM and CRC groups. On the basis of the clinicopathological characteristics of the enrolled patients, it was found that circ_0115744 was highly associated with liver metastasis (Table 3). A positive correlation of circ_0115744 expression was also observed in tissues samples when compared with the matched plasma samples (Figure 3B). Next, we conducted the loss- and gain-of-function assay to detect the endogenous expression of the circRNA circ_0115744 in CRC cell lines. DLD1 was selected as the cell model based on the medium expression level (Figure 3C). Next, DLD1 was treated with a circ_0115744-overexpressing lentivirus vector and two independent shRNA lentivirus vectors. The infection efficiency was measured by RT-PCR (Figure 3D, 3E). The Transwell assay was conducted to measure the function. The results showed that increased circ_0115744 expression promoted invasion of CRC cells, while decreased circ_0115744 expression suppressed cell invasion (Figure 3F); this finding indicated that circ_0115744 was highly associated with CRC metastasis.

**Table 3:** The clinicopathological characteristics of patients with CRC according to circ_0115744 expression.

		**circ_0115744^high^**	**circ_0115744^low^**	***p* value^a^**
	**n**	**n=40**	**n=40**	
**Age(years)**				
<60	32	18	14	0.361
≥60	48	22	26	
**Gender**				
Male	50	24	26	0.644
Female	30	16	14	
**Tumor diameter (cm)**				
<5cm	21	12	9	0.446
≥5cm	59	28	31	
**Pathologic type**				
ADC	75	37	38	0.644
MADC	5	3	2	
**Metastasis**				
Positive	40	38	2	<0.001
Negative	40	2	38	
**T stage**				
T1	10	4	6	0.575
T2	26	15	11	
T3	44	21	23	
**Differentiation degree**				
Highly	10	4	6	0.546
Moderately	58	23	25	
Poorly	12	13	9	
**Primary tumor site**				
Colon	39	21	18	0.502
Rectum	41	19	22	

a: Chi-square test; ADC: Adenocarcinoma; MADC: Mucinous adenocarcinoma.

### Identification of miRNA candidates that can bind to circ_0115744

**Figure 4:**
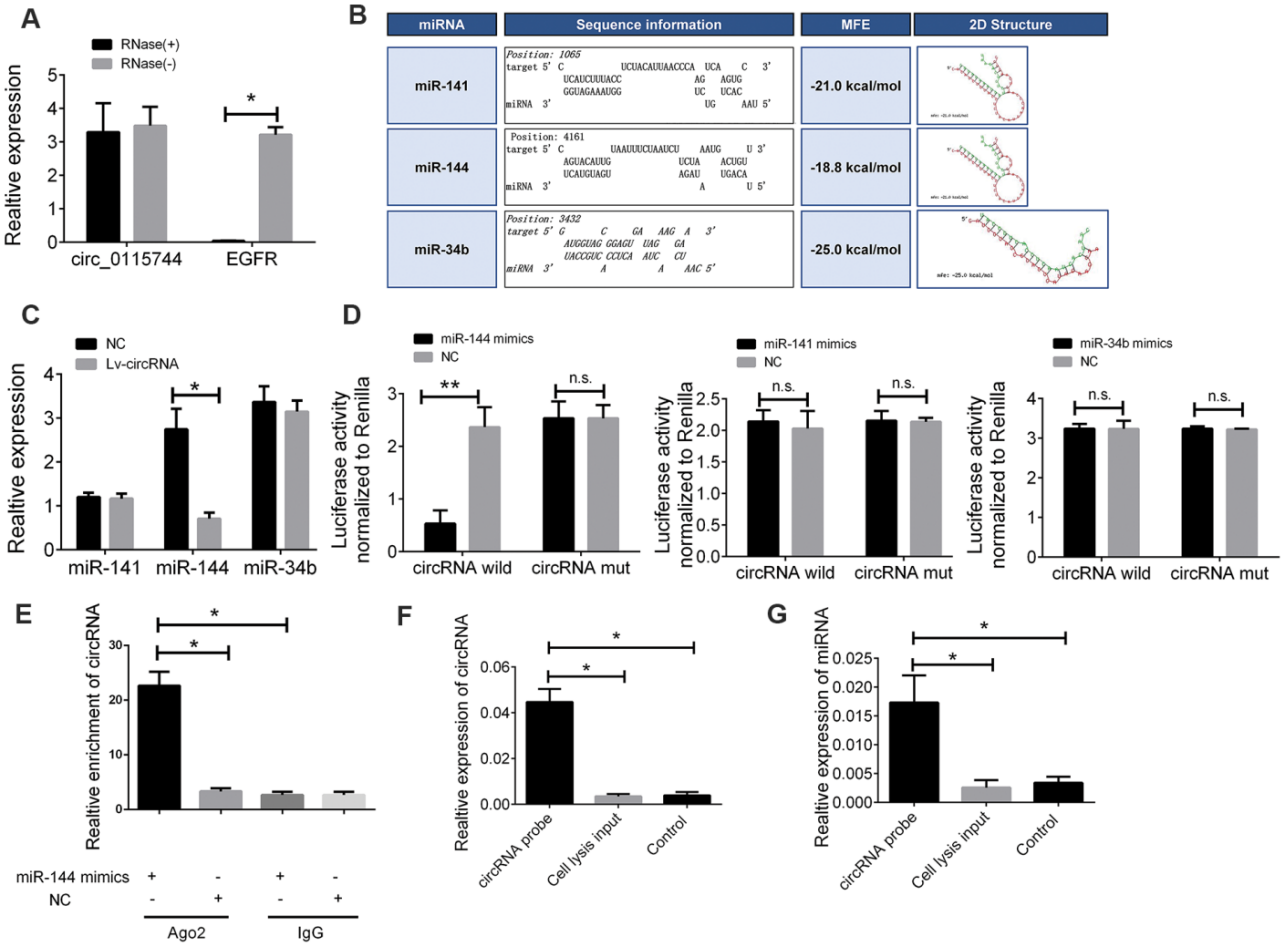
**The circ_0115744 could be bound by miR-144.** (**A**) The PCR analysis confirmed that circ_0115744 resisted to RNase R digestion. (**B**) Potential miRNA prediction. (**C**) Relative expression of miRNA in cells treated with circ_0115744 overexpression lentivirus. (**D**) Dual-luciferase reporter assay in cells treated with circ_0115744 and candidate miRNA mimics. (**E**) RIP analysis showed that circ_0115744 was abundantly pulled down by anti-Ago2 antibodies when transfected with miR-144 mimics, compared to the NC or IgG group. (**F, G**) Biotin-coupled probe pull down assay confirmed that miR-144 was effectively enriched by circ_0115744. Data were presented as plot of the mean with SEM. *Indicated P < 0.05, ** indicated P < 0.01, n.s. indicated no significant.

As there are few reports on the detailed mechanism of circ_0115744 in human cells, we next determined whether circ_0115744 could be digested by RNase R. By using EGFR as a control, we found that circ_0115744, instead of EGFR, can resist digestion by RNase R (Figure 4A). Given that circRNAs may act as competing endogenous RNAs (ceRNAs) of miRNAs to regulate mRNA expression, we next assessed the potential targets of circ_0115744 through a ceRNA-dependent mechanism. Furthermore, by using the bioinformatics tool Miranda, we predicted and screened the top three possible sponge miRNAs, including miR-141, miR-144, and miR-34b (Figure 4B). The subsequent qRT-PCR analysis showed that the upregulation of circ_0115744 decreased the expression of only miR-144 but not of miR-141 or miR-34b (Figure 4C). Next, the dual luciferase reporter assay was conducted to determine whether circ_0115744 could directly bind to the miRNA candidate. As shown in Figure 4D, we found that only the luciferase intensity of miR-144 was significantly suppressed. The RIP analysis also showed that more circ_0115744 was pulled down by anti-Ago2 antibody when transfected with miR-144 mimics in DLD-1 cells as compared to the miR-144 control group and the IgG group (Figure 4E). A biotin-coupled circ_0115744 probe was also designed for the pull-down assay, and the results showed that miR-144 was effectively enriched by circ_0115744 (Figure 4F).

### miR-144 directly targets EZH2

**Figure 5:**
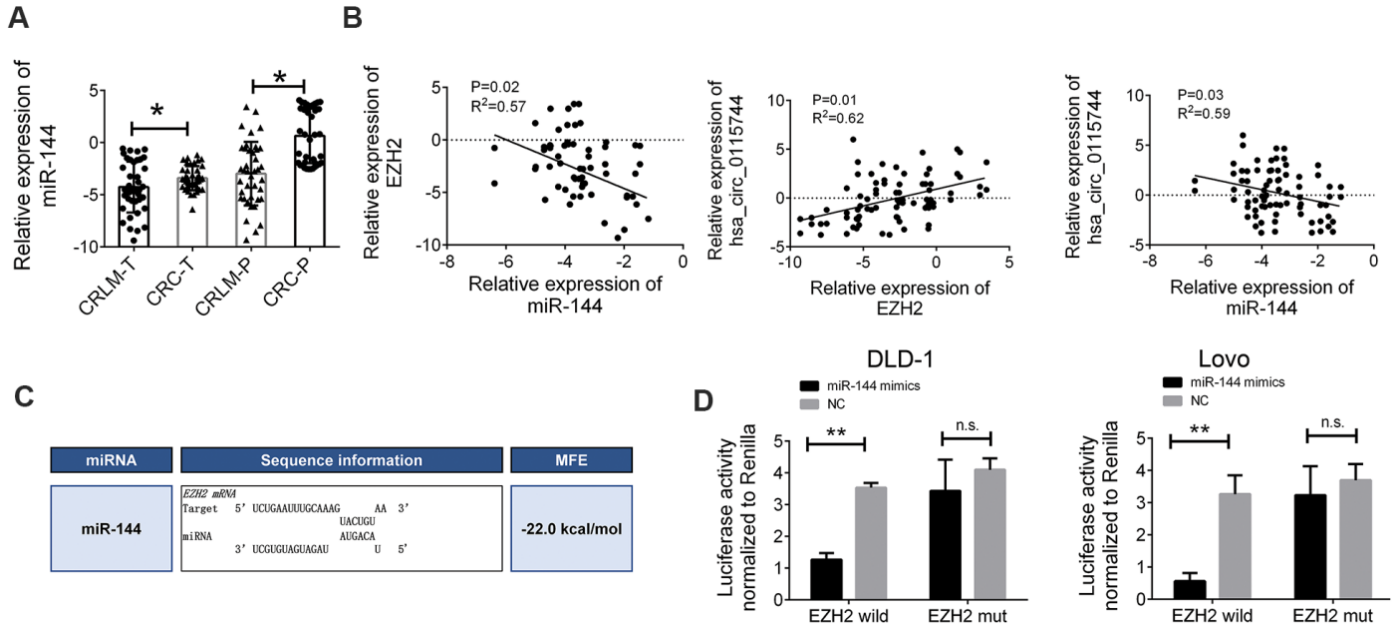
**miR-144 directly targeted EZH2** (**A**) Relative expression of miR-144 in tissues samples of CRLM and CRC. (**B**) Pearson correlation analysis for circ_0115744/miR-144/EZH2 in tissues. Data were presented as plot of the mean with SD with log-transformed. (**C**) Detailed binding site for miR-144 and EZH2. (**D**) Dual-luciferase reporter assay in cells treated with EZH2 3’UTR and candidate miR-144 mimics. Data were presented as plot of the mean with SEM. *Indicated P < 0.05.

To further elucidate the regulatory relationship between circ_0115744 and miR-144, the expression levels of miR-144 in the tissues samples of patients with CRLM and CRC were also determined. We found that miR-144 expression was downregulated in tumor tissues of both the CRLM and CRC groups (Figure 5A). miR-144 has been identified as a tumor suppressor in CRC, and many studies have reported that EZH2 is the main target gene of miR-144 [15, 16]. Therefore, we speculated that circ_0115744 may regulate the metastatic process through the miR-144/EZH2 pathway. Pearson’s correlation analysis was also used to confirm this finding. The results showed an inverse correlation between EZH2 and miR-144, a positive correlation between circ_0115744 and EZH2, and an inverse correlation between miR-144 and circ_0115744 (Figure 5B). The detailed binding site for miR-144 on EZH2 was also predicted (Figure 5C). A subsequent dual luciferase reporter assay indicated that miR-144 could directly bind to EZH2 (Figure 5D).

**Figure 6:**
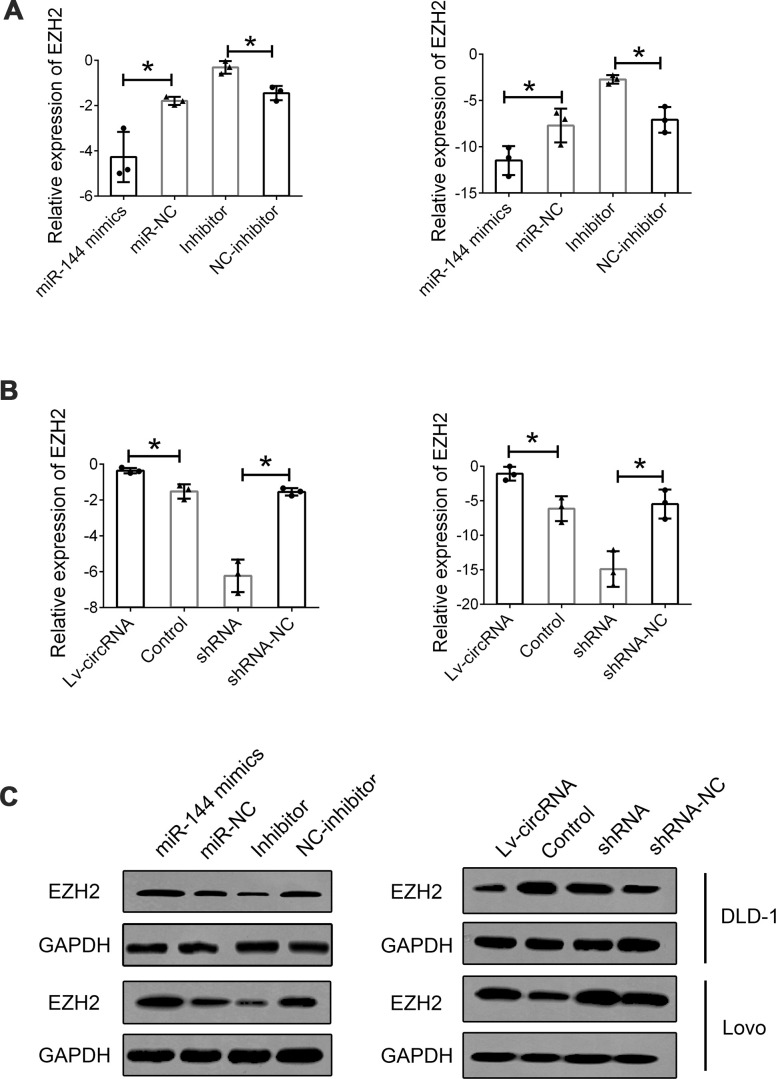
**Interaction between circ_0115744, miR-144 and EZH2.** (**A**) Relative mRNA expression of EZH2 in cells treated with miR-144 mimics or inhibitor. (**B**) Relative mRNA expression of EZH2 in cells treated with circ_0115744 overexpression or shRNA lentivirus. (**C**) Protein level of EZH2 in cells with different treatment. Data were presented as plot of the mean with SEM. *Indicated P < 0.05.

We further investigated the interaction between circ_0115744, miR-144, and EZH2. First, we detected the expression of EZH2 in cells treated with miR-144 mimics and an miR-144 inhibitor. The expression of EZH2 was suppressed following miR-144 overexpression, while EZH2 expression was upregulated through the inhibition of miR-144 (Figure 6A). Furthermore, the increased level of circ_0115744 also promoted the expression of EZH2, while the suppression of circ_0115744 expression downregulated EZH2 expression (Figure 6B). The protein level of EZH2 was also confirmed to be consistent with its mRNA level mentioned above (Figure 6C).

## DISCUSSION

With the improvement in economic well-being and living standards, the lifestyle of many people is becoming increasingly westernized. Subsequently, the incidence and number of deaths due to CRC have also significantly increased [17]. In China, CRC is included in the top five causes of death due to cancer [18]. Similarly, CRC has the third highest incidence among all cancers, and it also ranks fifth in cancer-related mortality in the USA in both males and females [19]. Thus, CRC is a significant threat to human health worldwide. One primary reason for such high morbidity and mortality of CRC is the lack of effective early diagnosis [1]. Therefore, it is quite important to seek new early tumor diagnostic markers. With the development of high-throughput sequencing technology, various human noncoding RNAs were found and identified as important regulatory factors in human malignant tumors. Increasing evidence has shown that circRNAs play a crucial role in carcinogenesis, cancer progression, and clinical outcomes of various human cancers. For example, researchers found that the circRNA circCCDC9 functions as a tumor suppressor molecule in inhibiting the progression of gastric cancer through the miR-6792-3p/CAV1 axis; this provided a useful biomarker and therapeutic target for patients with gastric cancer [20]. Higher plasma levels of exo-hsa_circRNA_0056616 in these patients also suggested that circRNA is a potential biomarker for lymph node metastasis prediction in lung adenocarcinoma [21].

Regarding CRC, we noted that hsa_circ_0082182, hsa_circ_0000370, and hsa_circ_0035445 were significantly increased in plasma samples of patients with CRC [22]. It was not detected in this study because we enrolled the liver metastasis group. However, when differentially expressed circRNAs in the CRC group were compared with those of the control group, we found that hsa_circ_0082182 and hsa_circ_0000370 but not hsa_circ_0035445 were present in the candidate list. In another study, circ-CCDC66, circ-ABCC1, and circ-STIL were defined as candidate biomarkers for patients with CRC [7]. In the present study, we did not observe a significant difference in our data. Based on these results above, we could find that many of the studies only focused on the early detection of CRC, although the microarray was also used, the statistical methods or predicting model was not diverse enough, most importantly, the liver metastasis was not enrolled.

EZH2 was found to participate in histone methylation, which regulates transcriptional repression. Increasing evidence has indicated that EZH2 is associated with cell proliferation, migration, and invasion by increasing the expression of the Wnt pathway inhibitors sFRP1 and DKK3 [23]. Furthermore, low miR-144 was found to upregulate EZH2 [16, 24]. In accordance with previous studies, we found that EZH2 was significantly upregulated in CRC tissues and cell lines. Knockdown of EZH2 inhibited cell proliferation, invasion, and metastasis of CRC cells. Moreover, cell proliferation and invasion ability stimulated by miR-144 inhibition could be reversed by knockdown of miR-144; this suggests that EZH2 is essential for mediating the biological effects of miR-144 in CRC cells. It was also found that mutations or aberrant upregulation of EZH2 occur frequently in human cancers [25]. In conclusion, in the present study, we identified that circ_0115744 could be a potential biomarker to predict liver metastasis of CRC. We also outlined the function of circ_0115744 during the metastatic process. circ_0115744 was identified as a tumor-promoting noncoding RNA in CRC through the miR-144/EZH2-dependent mechanism. Thus, these results suggest that circ_0115744 might be useful as a diagnostic marker in patients with CRLM and that silencing of circ_0115744 could serve as a new attractive therapeutic approach for CRLM.

## MATERIALS AND METHODS

### Patient samples

Samples, including plasma samples, tumor tissues, corresponding adjacent tumor tissues, and normal tissues, were obtained from patients with CRC from 2003 to 2015. Patients with CRC were diagnosed on the basis of surgical and pathological findings in the Second Affiliated Hospital of Soochow University and Suqian Hospital Affiliated to Xuzhou Medical University. After sample collection, liquid nitrogen was frozen and transported, and stored in a -70° C deep cryogenic refrigerator. Written consent was obtained from the patients enrolled in this study. The clinical stage was based on the 8th edition of the International Union Against Cancer (UICC) on the Tumor-Node-Metastasis (TNM) staging system. All experiments were performed in compliance with government policies and the Helsinki Declaration. All subjects were informed about the study and provided their consent prior to specimen collection. All experiments were approved by the ethics committee of the Second Affiliated Hospital of Soochow University.

### Risk score analysis

We performed the risk score analysis to determine the predictive ability of the candidate plasma circRNA. In brief, the upper 95% reference interval (95% CI) of each circRNA value in the control group was used as the cutoff value for the expression level of certain circRNAs. If the expression of circRNA in this sample was higher than the 95% CI value, we assigned as 1, and if lower than the 95% CI value, we assigned as 0. The risk score function (RSF) to predict the CRLM group was defined according to a linear combination of the expression level of each circRNA. For example, the RSF for sample i using information from three circRNAs was given as follows: rsfi=Σ3j-1Wj.sij. In this equation, sij is the risk score for circRNA j on sample i, and Wj is the weight of the risk score of circRNA j. The risk scores of the three circRNAs were calculated using the weight by the regression coefficient derived from the univariate logistic regression analysis of each circRNA. The samples were ranked according to their RSF and then divided into a high-risk group that represented patients with CRLM and a low-risk group that represented the predicted control individuals. Frequency tables and receiver operating characteristic (ROC) curves were then used to evaluate the diagnostic effects of the profiling and to find the appropriate cutoff value, and then to validate the procedure and cutoff values in the next validation sample set.

### CRC cell lines and cell culture

CRC cell lines, including DLD-1, HCT 116, HT 29, LoVo, Caco-2, and normal colonic mucosal FHC cells, were purchased from the Cell Bank of Type Culture Collection of the Chinese Academy of Sciences (Shanghai, China), and were cultured and stored according to the guidelines of the cell bank. The culture medium used was Dulbecco’s modified Eagle’s medium (DMEM; Winsent, Quebec, Canada) containing 10% fetal bovine serum (FBS), 100 U/ml penicillin, and 100 μg/ml streptomycin. All the cell lines were incubated in a 5% CO_2_ humidified incubator at 37° C.

### RNA isolation and real-time quantitative PCR

TRIzol reagent was used to isolate RNA from colorectal tissues or cell lines according to the manufacturer’s protocol. Next, 1 μg RNA was used to synthesize cDNA, followed by gene expression analysis on the ABI 7900 qPCR system. Relative expression was normalized to that of GAPDH. The primers used for GAPDH and circ-0115744 were as follows: GAPDH: forward: 5ʹ-GGAGCGAGATCCCTCCAAAAT-3ʹ, reverse: 5ʹ-GGCTGTTGTCATACTTCTCATGG-3ʹ; circ-0115744: forward: 5ʹ- TAGCAAATTCCTGCCCCGAG-3ʹ, reverse: 5ʹ-GCAGTGCAAATCTTGGCTCC-3ʹ.

### Transwell assay

Cell invasion was determined using Millicell cell culture inserts (24-well insert, 8 μm pore size). Cells were cultured in 200 μl serum-free medium in the upper chamber for the migration assay and in 200 μl medium supplemented with 10% FBS for the invasion assay. After 48 h of incubation, the cells on the lower side of the membrane were fixed, stained with crystal violet, and subjected to cell count.

### RNA immunoprecipitation assay

RNA immunoprecipitation (RIP) assay was performed according to a previously reported protocol. The Magna RIP RNA-Binding Protein Immunoprecipitation Kit (Millipore, CA, USA) was used, and the assay was conducted according to the manufacturer’s instructions. As the negative control, the IgG antibody was incubated in the suspension of magnetic beads with rotation for 30 min. After washing with ice-cold RIP wash buffer, the immunoprecipitated RNA was purified and detected by qRT-PCR.

### Bioinformatics analysis

The sequence of circ_0115744 was obtained from circbase (http://www.circbase.org), and TargetScan (http://www.targetscan.org/), and miRanda were used to predict the binding sites between circ_0115744 and miRNAs, and the potential target mRNAs of miR-144.

### Dual-luciferase reporter assay

The full-length sequence of circ_0115744 predicted to interact with miRNA or the mutated sequence with the predicted target sites was inserted into the *Hin*dIII and *Sac*I sites of the pMIR-REPORT luciferase vector (GenScript, Nanjing, China). The CRC cell lines cultured in 24-well plates were co-transfected with pMIR-REPORT vectors containing either the wild-type or mutated segments along with the control vector, and the pRL-TK vector containing Renilla luciferase was used for normalization. The CRC cells were co-transfected with the precursor microRNA mimics and the control group. Assays were performed to determine the gene expression level.

### Western blotting assay

Proteins were extracted from cells and tissues according to the manufacturer’s protocol (KeyGEN BioTech, Jiangsu, China). In brief, after extraction with RIPA buffer with protease inhibitor and phosphatase inhibitor cocktails (Pierce Biotechnology, Rockford, IL, USA) and quantification with a BCA kit (Thermo Fisher Scientific, Waltham, MA, USA), proteins from cell lysates or tissue lysates were equally loaded onto each well of SDS-PAGE. After electrophoresis, the proteins were transferred onto a membrane, blocked with 5% non-fat milk in PBST for 1 h, and then incubated with diluted primary antibodies at 4° C overnight. The protein expression levels were detected by ECL Plus (Millipore, Billerica, MA, USA) with a bio-imaging system.

### Statistical analysis

All statistical analyses were performed using SPSS 13.0 software (Chicago, IL, USA) and GraphPad Prism software (La Jolla, CA, USA). The chi-square test was used to analyze the correlation between circRNA expression levels and clinical features of the patients. Student’s t-test (two-tailed) was used to compare the results between two groups. Pearson’s correlation analysis was performed to determine the correlation between the expression of circRNA, miRNA, and mRNA. All confidence intervals (CIs) were estimated at the 95% confidence level. All data are presented as mean ± standard error of mean (SEM), and differences were considered to be statistically significant at P < 0.05.
